# Laparoscopic Single-Incision Triangulated Umbilical Surgery Adrenalectomy for Large (>5 cm) Tumors of the Adrenal Gland: Initial Experience with 16 Cases

**DOI:** 10.1155/2022/5966530

**Published:** 2022-09-28

**Authors:** Guang Feng Zhu, Xiao Liang Dou, Feng Qi Yan, Xiao Peng Chen, Qi Sheng Tang, Fan Liu, He Wang, Bo Zhang, Yong Wang

**Affiliations:** Department of Urology, Second Affiliated Hospital, Air Force Medical University, Xi'an 710038, Shaanxi, China

## Abstract

*Background and Objectives*Laparoscopic single-incision triangulated umbilical surgery (SITUS), which enables the extraction of intraabdominal specimens through a single umbilical incision, has yet to be used to perform adrenalectomy. We have modified SITUS to enable extraction of large (>5 cm) adrenal masses with optimal cosmetic outcomes and investigated efficacy and safety. *Methods*. In this retrospective study, we analyzed data of 16 patients with adrenal tumors >5 cm who had undergone adrenalectomy by SITUS between October 2015 and April 2018. Two C-shaped incisions were made around the umbilicus and sutured centripetally. After extracting the specimen, we evaluated these patients' operative/postoperative data. *Results*. SITUS was performed in all 16 patients without conversion to laparoscopic or open surgery. The mean operation time was 75.31 ± 21.54 min (intraperitoneal time 41.94 ± 17.57 min; incision suturing time 33.38 ± 6.34 min). The estimated median blood loss was 57.5 mL (range 30–610 mL). Drainage time and duration of hospital stay were 55.69 ± 12.92 h and 3.94 ± 0.90 d, respectively. After surgery, all incisions were hidden under the umbilicus. Three patients developed keloid diathesis, resulting in enlargement of their scars. *Conclusions*. SITUS is a safe and feasible procedure for removing large adrenal tumors. In addition to its cosmetic advantages, SITUS facilitates functional recovery, particularly in patients with large adrenal tumors.

## 1. Introduction

Since the introduction of laparoscopic adrenalectomy (LA) in 1992 [1], this has become a standard procedure for performing adrenalectomy [2]. Poor cosmetic outcomes [3] and incision-associated risks of bleeding, infection, and trocar-site hernias have resulted in research efforts focusing on reducing the number of surgical ports [4]. Novel techniques, such as laparoendoscopic single-site surgery (LESS), natural orifice transluminal endoscopic surgery (NOTES), and laparoscopic single-incision triangulated umbilical surgery (SITUS), have proved advantageous for patients with adrenal disease [5–7]. Because of the limited availability of appropriate laparoscopic devices and technical challenges, there are currently obstacles to performing pure natural orifice transluminal endoscopic upper urinary tract surgery [8, 9]. LESS requires a shorter incision, but has disadvantages related to lack of triangulation and collisions between instruments that are challenging for most urologists [10, 11]. Instruments designed specifically for LESS, including flexible endoscopes and articulating graspers, have not substantially reduced these difficulties [12]. LESS may be associated with more severe early postoperative pain [13] because it increases stress on port-site tissue and may confer an increased risk of trocar-site herniation [14, 15].

SITUS [5], a novel, minimally invasive procedure, has many advantages for both surgeons and patients. After establishing a stretchable “C”-shaped incision around the umbilicus, the viewport and operating instruments are inserted into the abdominal cavity. SITUS does not require special laparoscopic instruments or particular laparoscopic operative techniques. Learning curves for establishing the ports and closing the incision are therefore short [16]; additionally, postoperative cosmetic outcomes are better than those after traditional procedures. SITUS has been used for radical nephrectomy, partial nephrectomy, radical cystectomy, and pyeloplasty [5, 16–18]. However, to the best of our knowledge, there are no published reports on performing this type of procedure for adrenalectomy.

In this retrospective study, we analyzed clinical data on patients who had undergone SITUS adrenalectomy for large adrenal tumors (diameter > 5 cm) in our medical center between October 2015 and April 2018. Our aim was to summarize our initial experience with SITUS adrenalectomy.

## 2. Materials and Methods

### 2.1. Patient Selection and Outcome Measurements

After obtaining the approval of the Institutional Review Board of Second Affiliated Hospital, Air Force Medical University, we retrospectively reviewed the medical records of all patients who had undergone SITUS adrenalectomy. After being informed of its advantages and disadvantages, patients requiring adrenalectomy between October 2015 and April 2018 were invited to choose whether to undergo SITUS. Written informed consent for the procedure was required by the Institutional Review Board, which waived the need for consent to inclusion in the study because it was a retrospective study of anonymized data.

Eligibility criteria included an indication for adrenalectomy, computed tomography evidence of a large mass (>5 cm), and appropriate medical status for surgery (American Society of Anesthesiologists Physical Status classification 1 or 2). The patients were informed of the benefits and disadvantages of SITUS and the possibility of requiring conversion to conventional laparoscopy. Exclusion criteria comprised height >190 cm or <150 cm, American Society of Anesthesiologists score ≥ III, presence of a bleeding disorder, body mass index (BMI) over 32 kg/m^2^, and presence of extensive tumors that infiltrated adjacent organs and required en bloc resection.

From October 2015 to April 2018, SITUS was performed on 16 of 23 patients requiring adrenalectomy for large adrenal tumors (diameter > 5 cm) in the Department of Urology, Second Affiliated Hospital. Prior to surgery, each patient underwent computed tomography scanning to determine the characteristics and sizes of their tumors. Preoperative examination, preparation, anesthesia management, and intraoperative invasive monitoring were performed as reported previously [19].

Relevant patient characteristics and perioperative data were collected by an investigator who was not involved in performing the surgeries. Patient characteristics, which included age, sex, BMI, tumor size, and tumor side, are shown in Table 1. Collected perioperative data included estimated blood loss, operative time, intraperitoneal operation time, incision cutting and suture times, number of assistant ports, blood transfusion, hospital stay time, drainage time, Patient Scar Assessment Questionnaire (PSAQ) score (range 34–136) [20], visual analog scale (VAS) scores for pain, and pathological findings. Three of the 16 patients had previously undergone abdominal surgery (one hysterectomy, one appendectomy, and one laparoscopic cholecystectomy); these three patients all had left-sided adrenal tumors.

### 2.2. Operative Procedure

All patients provided informed consent for SITUS and understood the risks of requiring conversion to traditional LA or open surgery.

**Figure 1:**
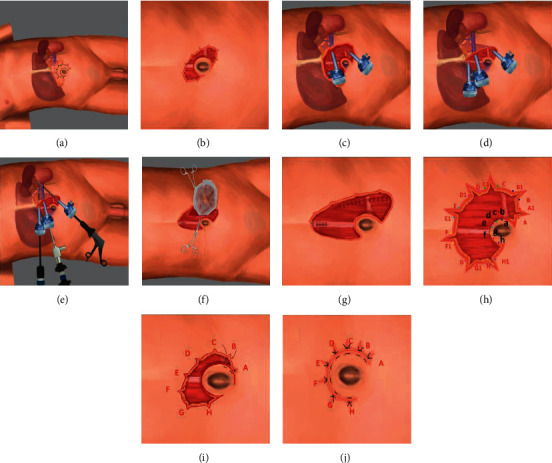
Schematic diagram of the left SITUS incision. (a) Patient's position: the patient was positioned in a standard 70° recumbent position, padding the waist high. The incision line was made on the abdominal wall and serrated notches were added to increase the tension of the outer rim. (b) A cut was made along the line as shown. The skin and subcutaneous tissues were then removed. The arc back of the umbilicus incision was oriented toward the operation field. (c) A trocar was inserted into the viewport at the midpoint of the C-shaped incision as far as possible from the umbilicus. The right-hand operative port was moved vertically down the viewport as low as possible. (d) The left-hand operative port was then placed at the highest position of the midline of the torso. (e) Diagrammatic sketch showing the relationship between the incision, the trocars, and the tumor. (f) The viewport and the right-hand operative port were then connected and the sides were drawn out to remove the specimen. (g–j) show how the incision was sutured. The side incisions around the C-shaped main incision were made by removing the skin and subcutaneous tissue between the paired points A and A1, B and B1, and so on; this widened the distance between these ports. The increased serrated side incision could also be sutured into the umbilicus by suturing the subcutaneous tissue by the rule of suturing three points—A, A1, and a—together (h, i), thus forming a single point (A) as shown. The skin between these points (A–H) was then sutured intradermally with VICRYLTM Rapide (ETHICON).

**Figure 2:**
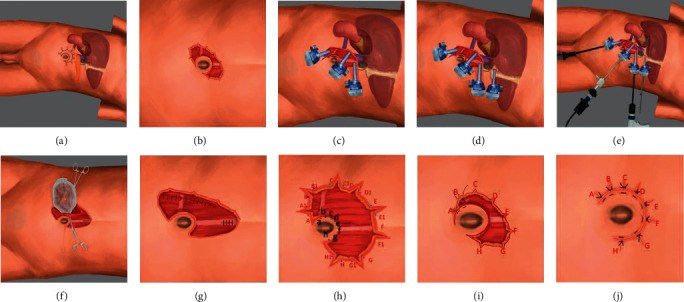
Schematic diagram of the right SITUS incision. (a–j) show the same meaning as Figure 1. (d, e) Show the assistant port during a right-sided procedure to retract the liver lobe and expose the adrenal tumor.

The patient was positioned in a lateral decubitus position with the affected side elevated by 70° (Figures 1(a) and 2(a)). The general principles for SITUS umbilical incisions have previously been described [5]. We modified the published procedure as follows: First, after incision of the skin and subcutaneous tissue, we made seven to nine serrated incisions (depending on the tumor size) along the outer rim to increase the ability of the umbilical incision to stretch on retraction (in Figures 1(a) and 2(a)). Second, we aimed the arc at the top of the C-shaped umbilical incision at 45° cephalad (Figures 1(b) and 2(b)) rather than in the reported direct cephalad direction [5]. We then added side incisions around the C-shaped outer rim to lengthen the umbilical incision, thus enabling spaces of >4 cm between trocars (Figures 1(c)–1(e) and 2(c)–2(e)). These variations resulted in trocar settings that are more like those used in conventional LA and enabled our instruments to move more freely than when performing SITUS as originally reported [5]. We also made the following changes to the trocar layout. After removing the skin and subcutaneous tissue, we used thyroid hooks to extrude the midpoint of the arc-shaped incision in the direction of the surgical area. We then inserted a pneumoperitoneum needle into the abdominal cavity at the cross-point of the lateral border of the rectus abdominis and the lower rim of the umbilicus. When the pneumoperitoneal pressure had reached 15 mmHg, we inserted the view trocar into the abdominal cavity at the midpoint of the C-shaped incision as far away from the umbilicus as possible. The inferior operation port was inserted vertically down the viewport into the abdominal cavity as inferiorly as possible. The superior operation port was established at the most cephalad position of the midline of the torso when the outer rim of the incision was retracted. For the left side, the inferior operation port was for the right hand and the superior one for the left hand, whereas the opposite settings were used for the right side. For right adrenalectomy, an extra 5 mm trocar was added at the intersection of the subcostal and midclavicular lines to enable retraction of the liver with a fan-shaped endoretractor (Figure 2(e)). The additional trocar incision was utilized for drainage after the procedure. Intraabdominal operative procedures were performed as previously described [21, 22]. The abdominal wall was cut open between the viewport and inferior operation port to enable extraction of the specimen (Figures 1(f) and 2(f)). The peritoneum and rectus abdominis sheath were sutured in a routine manner (Figures 1(g) and 2(g)). To close the incisions, the modified serrated side incisions were sutured into the umbilicus by suturing the subcutaneous tissue at three points—A, A1, and a—together (Figures 1(h), 1(i), 2(h), and 2(i)), thus creating a single point at A (Figures 1(i) and 2(i)). This procedure resulted in the shortening of the main incision. The skin surrounding the main incision between these points (A–H) could then be sutured with the inner rim intradermally with VICRYL™ Rapide (ETHICON) (Figures 1(j) and 2(j)).

### 2.3. Statistical Analysis

All data were analyzed using SPSS 18.0 (SPSS; Chicago, IL, USA) for Windows.

Continuous variables are expressed as mean ± SD. Age, BMI, and blood loss are expressed as median (interquartile range).

## 3. Results

**Figure 3:**
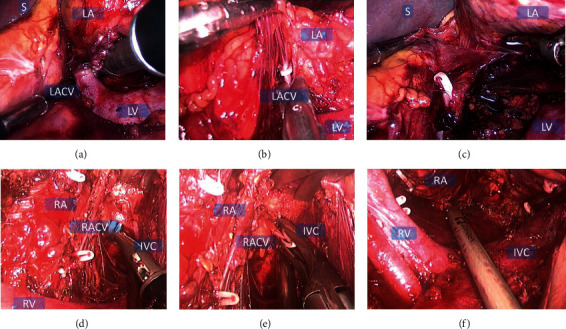
Intraperitoneal operation charts for the SITUS technique. (a) The left central adrenal vein and the left renal vein. (b) Cutting off the central adrenal vein. (c) Cutting off the connective tissue and blood vessels between the left adrenal tumor and the abdominal aorta. (d) The right central adrenal vein and the inferior vena cava. (e) Cutting off the right central adrenal vein. (f) The space between the right adrenal tumor and the Psoas major. *S* = spleen; LR = left kidney; LA = left adrenal gland (tumor); LACV = left adrenal gland central vein; LV = left renal vein; RA = right adrenal gland (tumor); RV = right renal vein; IVC = inferior vena cava; RACV = right adrenal gland central vein; RK = right kidney.

**Figure 4:**
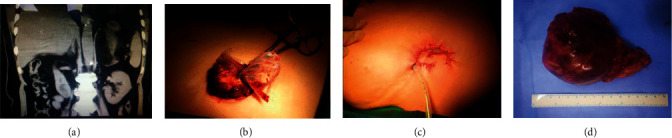
(a) Computed tomography scan of the adrenal tumor. (b) Image showing the specimen being removed from the abdominal cavity. (c) Image showing the sutured incision, with the drainage tube aside. (d) Image showing the resected tumor specimen.

All 16 SITUS procedures were accomplished without conversion to traditional LA or open surgery. The layout of the viewport and operative ports was modified such that the endoscope and instruments formed an upright isosceles triangle (Figure 3), enabling the instruments to be moved freely within the abdominal cavity with minimal risk of collision. Large adrenal tumors could easily be extracted and the incisions sutured so as to be hidden in the umbilicus (Figure 4 is a CT image showing an 8.3 cm left adrenal tumor).

Perioperative and postoperative characteristics are summarized in Table 2. The mean operative time was 75.31 ± 21.54 min. All adrenal masses were excised en bloc without mortality or major morbidity. All hemodynamic events were resolved by drug treatment without unacceptable fluctuations in blood pressure. No additional trocars were required during any of the procedures. No complications classified as Grade III or higher on the Clavien–Dindo system occurred. Six patients had minor perioperative complications. One patient with a large (8.3 cm) left adrenal mass with an abundant blood supply had an intraoperative blood loss of about 610 mL. This patient was transfused with two units of red blood cells. Five of the 16 patients needed nonsteroidal analgesics on the day of surgery, three on a postoperative Day 1, and none from postoperative Day 2 onwards.

Patients were followed up for at least 12 months, during which there were no perioperative or postoperative complications. No patients with malignant tumors developed local recurrences or port-site metastases within those 12 months.

**Figure 5:**
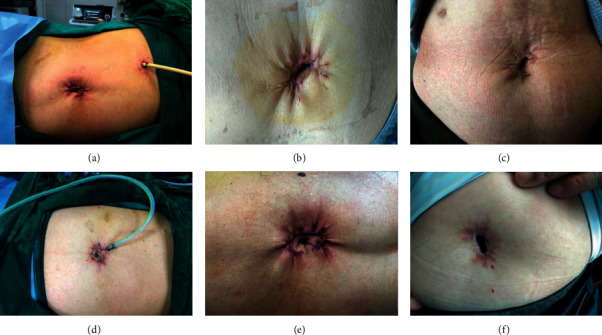
(a–c) show images for the right-side SITUS technique being used for adrenalectomy immediately after surgery, and at 1 week and 1 year after surgery, respectively. The drainage tube may be placed under the costal margin for an auxiliary incision. (d–f) show images of the wounds for the left-side SITUS technique being used for adrenalectomy immediately after surgery, and at 1 week and 1 year after surgery, respectively. The drainage tube may be placed in the periumbilical incision.

After the procedure, the incisions surrounding the SITUS surgical site were generally hidden in the umbilicus and the cosmetic outcomes were satisfactory. No patients developed port-site hernias or had chronic wound pain. The mean PSAQ scores were 54.81 ± 9.63 3 months and 47.06 ± 6.20 12 months after surgery (Table 2).  Figure 5 shows left- and right-sided photographs of the wounds immediately after the procedure, and then at 1 week and 12 months after surgery.

## 4. Discussion

Traditional laparoscopy usually requires three to five trocars, thus leaving three to five trocar scars. Furthermore, the specimen retrieval incision, which must be longer than the minimum diameter of the specimen, usually has a greater influence on the cosmetic outcome, especially in patients with large (>5 cm) tumors. Sufficiently long incisions may injure peripheral sensory and motor nerves, causing sensory disturbances in the skin and dysfunction of relaxation in the abdominal wall musculature [3, 23].

During the LESS procedure, all operative instruments and the laparoscope are inserted through multiple channels in a single port. However, each type of access has its own technical difficulties and limitations. First, the lack of triangulation results in intraoperative collisions between the instruments and laparoscope, creating the so called “chopsticks” or “sword-fighting” effect [10, 11]. Second, the direction of the laparoscope is limited by the direction of the instruments, which causes poor quality of vision, particularly when bleeding occurs. Third, surgeons require a special learning curve [7]. Fourth, the intraperitoneal operative time required is longer [24–26]. Fifth, special instruments, such as prebent or flexible instruments, are required [27, 28] and these tend to bend, especially when exerting counter-traction while dissecting large masses. Finally, the incision for removal of the specimen must be longer than the specimen's minimum diameter. This is a crucial point and neglecting it can result in incision-related adverse events [13, 14].

Nagele et al. were the first to report performing SITUS for nephrectomy in 2012 [5]. The ports for SITUS are relatively concentrated around the umbilicus. This has four main advantages. First, the trocars are arranged in a triangular pattern. SITUS involves a stretchable C-shaped incision around the umbilicus, which ensures triangulation of the laparoscopic procedure. Second, no special instruments are needed: SITUS requires only conventional laparoscopic instruments because of the triangulated layout of the instruments and endoscope. Furthermore, the triangulated layout of the trocars and the distances between trocars (≥4 cm) significantly reduce the operative difficulty, thus shortening the learning curve [16]. Third, on completion of the procedure, the incisions are sutured to the edge of the umbilicus, thus achieving satisfactory cosmetic outcomes. Fourth, Schoenthaler et al. assessed the learning curves required for SITUS, LESS, and conventional laparoscopy and concluded that SITUS is significantly easier to learn than LESS [16].

We have made several improvements to the SITUS procedure. First, we have added seven to eight side incisions on the outer rim of the umbilical incision to increase the stretched length of that incision to more than 12 cm. Second, we have rearranged the layout of the trocars such that they form an isosceles triangle with sides of 4 cm or more. Third, we have rotated the top of the C-shaped incision so that it points to the surgical area. Because our modified technique requires only standard operative instruments and endoscopes, experienced laparoscopic surgeons need no extra training beyond establishing the positions of the trocars.

Our improvements have many advantages. The isosceles triangle arrangement of the trocars, which is made feasible by the longer arc length, is markedly superior to the linear layout described previously. This modification allows the instruments to move freely intraoperatively with minimal risk of collision. The periumbilical incision facilitates anatomical closure in obese patients. The additional serrated side incisions can be sutured into the umbilicus in a centripetal fashion, thus achieving cosmetic outcomes that are close to equivalent to those achieved by previous SITUS procedures. This is because the side incisions resemble wrinkles in the periumbilical skin. Finally, specimen retrieval is facilitated, thus limiting operative trauma. The 12 cm-long incision enables retrieval of most specimens without lengthening the incision.

We acknowledge that our study has several limitations. First, there were only 16 cases in this preliminary study of SITUS adrenalectomy for adrenal gland tumors >5 cm. Larger prospective, randomized, double-blind, and controlled studies are needed to draw firm conclusions. Furthermore, most adrenal tumors are smaller than 5 cm and their retrieval does not require a 12 cm incision. Despite these limitations, we believe that our modified form of SITUS offers a valuable alternative for selected patients.

## 5. Conclusions

SITUS is a safe and feasible procedure for adrenalectomy. With our modified technique, the postoperative scar is fully integrated into the umbilicus and the cosmetic effect is good. With our modifications of SITUS, no specialized equipment is needed, only the standard instruments and endoscopes that have traditionally been used for laparoscopy. Thus, the procedure can be performed in most medical centers. A further large-scale, randomized, controlled study is needed to compare the effects of SITUS with those of traditional forms of laparoscopic and robotic laparoscopy. This will allow us to make further, more robust recommendations for the application of SITUS.

## Figures and Tables

**Table 1:** General characteristics of the patients.

Characteristics	All (*N* = 16)	%
Median age (range) (yr)	59.5 (34, 72)	—

Gender (%)	16	100
Male	10	62.5
Female	6	37.5

Median BMI (kg/m^2^) (IQR)	23.50 (21.45, 25.63)	—
BMI < 25	10	62.5
25 ≤ BMI ≤ 28	4	25
BMI > 28	2	12.5

Maximum tumor diameter (cm)	6.17 ± 3.996	—

ASA score		
1	7	43.7
2	9	56.3
3	0	0

Side (%)	16	100
Right	9	56.25
Left	7	43.75

Previous abdominal surgery (%)	3	18.75

**Table 2:** Intraoperative and postoperative outcomes.

Characteristics	Data
Estimated blood loss (median (range), ml)	57.5 (30–610)
Operative time (mean ± SD, min)	75.31 ± 21.54
Intraperitoneal operation time	41.94 ± 17.57
Incision cutting and suture time	33.38 ± 6.34
Adding assistant port	Right side 9 (56.25%)
Blood transfusion (%)	1 (6.25%)
Hospital stay time (d)	3.94 ± 0.90
Drainage time (h)	55.69 ± 12.92
PSAQ score at 3 m	54.81 ± 9.63
PSAQ score at 12 m	47.06 ± 6.20
Pathological findings	16 (100%)
Adrenocortical adenoma	4 (25.00%)
Pheochromocytoma	3 (18.75%)
Adrenocortical carcinoma	4 (25.00%)
Myelolipoma	3 (18.75%)
Adrenal nonfunctional adenoma	2 (12.50%)
VAS at 24 h (mean ± SD)	4.36 ± 2.57
VAS at discharge (mean ± SD)	0.94 ± 1.09
Complications	
Conversion to LA	0
Conversion to open surgery	0

## Data Availability

The data reported are available and are included in our database of adrenal tumors.
